# Effect of psychopharmacological combinations on psychopathological symptom burden and BMI trajectories in patients with eating disorders

**DOI:** 10.3389/fpsyt.2026.1750187

**Published:** 2026-01-26

**Authors:** Ernesta Panarello, Francesco Monaco, Valeria Di Stefano, Annarita Vignapiano, Roberta Zaffonte, Ilaria Pullano, Giulio Corrivetti, Luca Steardo, Michele Fornaro

**Affiliations:** 1 Department of Mental Health, ASL Salerno, Salerno, Italy; 2 European Biomedical Research Institute of Salerno (EBRIS), Salerno, Italy; 3 Psychiatric Unit, Department of Health Sciences, University Magna Graecia of Catanzaro, Catanzaro, Italy; 4 Department of Neuroscience, Reproductive Sciences, and Dentistry, Section of Psychiatry, University School of Medicine Federico II, Naples, Italy

**Keywords:** Anorexia Nervosa, Antipsychotics, BMI, mood stabilizers, Psychopharmacology, Real-worldstudy, Symptom burden

## Abstract

**Introduction:**

Eating disorders (EDs), particularly Anorexia Nervosa (AN), remain one of the most severe and treatment-resistant eating disorders, with high relapse rates and limited pharmacological options. While second-generation antipsychotics and antidepressants are commonly prescribed as adjuncts to nutritional rehabilitation, their real-world impact on weight restoration and psychopathological symptom severity remains unclear.

**Methods:**

We conducted a prospective, naturalistic observational study of 127 inpatients diagnosed with restrictive anorexia nervosa (AN-r), binge–purge anorexia nervosa (AN-bp), or bulimia nervosa (BN). BMI and weekly psychopathological symptom burden were systematically monitored throughout a ten-week inpatient treatment program. Psychopharmacological treatments were recorded in real time, and the Average Morbidity Index (AMI), adapted from the Life Chart Methodology, was computed weekly as a prospective measure of clinical severity. Generalized Linear Models assessed the associations between specific drug classes and changes in BMI and AMI.

**Results:**

Patients treated with mood stabilizers (e.g., Carbamazepine, Lithium) showed a smaller BMI increase compared to other groups (Coef. = –0.96 to –1.70; p< 0.05), suggesting a potential weight-stabilizing effect. Diazepam use was associated with greater weight gain (Coef. = +2.06; p = 0.02) but no clear benefit on AMI. Several antidepressants (e.g., Sertraline, Escitalopram) correlated with higher AMI scores, indicating less improvement in psychopathological symptom burden. Atypical antipsychotics (e.g., Olanzapine, Aripiprazole) were linked to greater reductions in AMI.

**Conclusions:**

Prospective monitoring of BMI and AMI revealed differential associations between psychopharmacological agents and both nutritional and symptomatic trajectories in an inpatient ED cohort predominantly composed of patients with AN. Mood stabilizers were associated with smaller BMI increases, while several antidepressants corresponded to less improvement in psychopathological symptom burden. Atypical antipsychotics showed the strongest prospective reductions in AMI. These findings highlight the value of prospective, real-world monitoring to inform pharmacological strategies in treatment-resistant eating disorders.

## Introduction

Eating disorders (EDs) are severe psychiatric conditions characterized by disturbances in eating behavior, body image, and weight regulation, and are associated with significant medical morbidity, functional impairment, and elevated mortality. Among EDs, Anorexia nervosa (AN) is a multifaceted and severe psychiatric disorder marked by deliberate dietary restriction, pronounced disturbances in body image, and an overwhelming fear of gaining weight (1, 2). It predominantly affects adolescent girls and young adult women and is associated with considerable medical morbidity, impaired functioning, and one of the highest mortality rates reported in psychiatry (3). While Bulimia Nervosa (BN) and other EDs are also associated with substantial psychopathological burden, inpatient treatment settings are predominantly occupied by individuals with AN, particularly restrictive and binge–purge subtypes. Although several psychotherapeutic interventions, especially cognitive behavioral therapy (CBT), are available, many patients do not achieve sufficient weight restoration during treatment, particularly in inpatient settings, and relapse remains common. While EDs comprise several diagnostic categories, the present sample is largely composed of patients with AN, reflecting the higher frequency of hospitalization observed for this condition in our clinical setting. The pharmacological management of EDs is often challenging and requires a nuanced, individualized approach. Inpatient programs for EDs, particularlyAN, usually emphasize medical stabilization, nutritional rehabilitation, and structured psychological support. Nonetheless, a substantial proportion of patients struggle to achieve and maintain meaningful weight gain Current treatment of EDs relies primarily on nutritional rehabilitation and structured psychotherapeutic interventions. However, a substantial proportion of patients, especially those with severe AN, fail to achieve sustained weight restoration or show persistent psychopathology despite intensive treatment (4). Some individuals show marked resistance to nutritional interventions, persistent anxiety related to eating, or cognitive rigidity that hinders engagement in treatment. Anxiety related to eating, cognitive rigidity, affective instability, and compulsive behaviors frequently interfere with treatment engagement and recovery, prompting the widespread use of adjunctive psychopharmacological strategies in routine clinical practice (5). These clinical complexities have encouraged clinicians and researchers to investigate pharmacological adjuncts as a means of supporting weight restoration and addressing the core psychopathological mechanisms that contribute to treatment resistance. Recent international treatment guidelines also address the use of pharmacological agents in EDs. The 2023 update of the World Federation of Societies of Biological Psychiatry (WFSBP) guidelines on the pharmacological treatment of EDs provides graded recommendations on medications such as atypical antipsychotics and antidepressants, highlighting limited but potentially supportive roles for these agents when used as adjuncts to nutritional and psychological interventions. These guidelines emphasize that no medication is formally approved for core features of anorexia nervosa and that pharmacotherapy should be individualized based on comorbidities, psychopathological symptom burden, and risk–benefit considerations (6).

Among the pharmacological agents most considered are second-generation antipsychotics, such as olanzapine, aripiprazole, and quetiapine, which may promote weight gain through metabolic mechanisms and reduce symptoms like obsessions, rigidity, and food-related anxiety (7). Specifically, olanzapine has been explored for its potential influence on affective dysregulation and dopaminergic alterations implicated in reward-related disturbances in AN (8). Therefore, the largest randomized controlled trial to date, conducted by Attia et al. (9), demonstrated that olanzapine was associated with significantly greater weight gain compared with placebo in adult outpatients with anorexia nervosa, although its effects on core eating disorder psychopathology were modest. This trial represents the strongest evidence supporting a pharmacological role for olanzapine in weight restoration, while underscoring the persistence of symptomatic burden despite treatment.

Nonetheless, findings remain inconsistent, and methodological limitations, including small samples, varying treatment protocols, and heterogeneity across studies, have obstructed firm conclusions about its efficacy in accelerating weight gain or improving overall outcomes (8, 10). In addition, randomized controlled evidence exists for other pharmacological agents not represented in the present cohort. In particular, Andries et al. (11) reported that dronabinol was associated with modest but significant weight gain in patients with severe and enduring AN. As no patients in the current study were treated with dronabinol, its effects could not be examined here; however, this evidence further highlights the heterogeneity of pharmacological strategies explored in AN Antidepressants, particularly selective serotonin reuptake inhibitors (SSRIs) such as fluoxetine and sertraline, are also routinely prescribed to individuals with EDs, especially those with AN (12). Their use is often driven by the presence of comorbid anxiety and depressive symptoms, which occur frequently in this population. However, their direct influence on weight restoration is uncertain (13, 14). Evidence suggests that their effectiveness may be diminished in severely underweight patients due to altered pharmacokinetics and reduced serotonin availability (15). Furthermore, randomized trials have not consistently demonstrated benefits for weight gain or relapse prevention when SSRIs are used during the underweight phase of illness (13, 14). Still, they remain widely administered in clinical settings, frequently initiated during inpatient treatment and continued thereafter. Despite the frequent clinical use of both antipsychotics and antidepressants in EDs, particularly AN, few studies have directly compared their impact on weight trajectories during hospitalization (16, 17). In addition, limited data exists on the combined use of these medications, the relative contribution of individual agents, or the ways in which different psychopharmacological combinations may influence the course of nutritional rehabilitation (18). The present study seeks to address these gaps by examining the association between antipsychotic and antidepressant treatments and changes in body mass index (BMI) in a real-world inpatient cohort of patients with EDs, predominantly with AN. By analyzing prospectively collected clinical data, we investigate whether specific medications or drug combinations are linked to more pronounced increases in BMI over the course of treatment. We also explore whether different pharmacological classes, such as first- versus second-generation antipsychotics or SSRIs versus other antidepressants, exhibit distinct patterns of association with BMI outcomes. In addition to assessing the magnitude of weight change, we consider the broader clinical significance of psychopharmacological interventions in a context where intensive nutritional and psychological treatment is already in place. By providing insights into real-world prescribing patterns and their relationship with anthropometric outcomes, this study may support more informed clinical decision-making in the acute management of AN. Moreover, understanding the practical effectiveness of commonly used medications may contribute to the development of more personalized and data-driven treatment strategies for a vulnerable and clinically complex population.

## Methods

### Study design

This was a prospective, naturalistic observational study conducted in a specialized inpatient program for EDs (AN and BN) admitted to a specialized clinical center. All clinical data, including BMI records and pharmacological prescriptions, were recorded prospectively as part of routine care across a structured ten-week inpatient treatment protocol. Data collection followed a predefined schedule to ensure standardized assessment across the entire observation period. The primary objective was to examine prospective associations between psychopharmacological treatments, BMI change (ΔBMI), and weekly morbidity trajectories measured through the AMI. Formal ethical approval was not required for this prospective, naturalistic observational study, as data were collected as part of routine clinical care and analyzed in fully anonymized form, in accordance with national regulations and institutional policies.

### Participants

A total of 127 patients were included in the analysis (mean age 18.7 ± 5.5 years, range 11–39 years). Socio-demographic data (age, gender, educational level, age of onset) and clinical variables (specific diagnosis, BMI at admission and discharge, pharmacological treatments) were systematically collected. Although the study included a small proportion of patients with BN, the results predominantly reflect the inpatient treatment course of individuals with AN, who constitute most of our sample.

### Pharmacological variables

Medication entries reflect all psychotropic agents prescribed at any point during the ten-week inpatient program. Because treatment adjustments, medication switches, or short titration periods occurred as part of routine care, some compounds appear more than once in descriptive tables; these entries represent distinct exposure windows rather than duplicated records. Polypharmacy was common in this real-world setting, and many patients received combinations of antidepressants, antipsychotics, mood stabilizers, or benzodiazepines over the course of treatment. For this reason, analyses were conducted based on exposure to each compound or pharmacological class rather than mutually exclusive medication groups.

### Variables

- BMI: BMI was recorded at the time of admission and again at discharge from our inpatient department. The primary nutritional outcome (ΔBMI) was defined as the difference between discharge BMI and baseline BMI.
- Average morbidity index (AMI): A composite index was calculated for each patient to capture the duration and severity of psychopathological symptoms throughout the observation period. The AMI was conceptually derived from the Life Chart Methodology originally developed for mood disorders (19) and adapted for this study based on weekly clinical chart entries. The Average Morbidity Index (AMI) was computed prospectively based on weekly documented symptom severity ratings in clinical charts. The AMI was conceived as a measure of overall psychopathological morbidity rather than a scale of ED –specific behaviors. Weekly symptom severity ratings reflected the presence and intensity of clinically relevant affective, anxiety-related, cognitive, and behavioral symptoms documented in routine clinical charts, including mood instability, anxiety, obsessive–ruminative thinking, emotional dysregulation, agitation, sleep disturbance, and behavioral dyscontrol. ED-specific behaviors (e.g., food restriction, purging) were not the primary target of the AMI, as these are often externally contained during inpatient treatment.The AMI was computed as:

$$AMI=\frac{\left(W_{1}\times 1)+(W_{2}\times 2)+(W_{3}\times 3\right)}{Total\;weeks\times 3}$$

where W_1_–W_3_ represent the number of weeks with psychopatological symptom severity grades 1–3, respectively. The AMI was monitored weekly as a secondary outcome to describe the overall morbidity and clinical burden but was not the primary endpoint.

Pharmacological treatments: Treatments were coded as binary indicators (0 = no; 1 = yes) for each main pharmacological class: antidepressants (SSRIs, SNRIs, TCAs, atypicals), mood stabilizers (Carbamazepine, Lithium, Valproate, Lamotrigine, Oxcarbazepine), typical and atypical antipsychotics, and benzodiazepines.

### Statistical analysis

- Descriptive analysis: Summary statistics (mean, standard deviation, range) were calculated for age, BMI at admission and discharge, ΔBMI, AMI, and the distribution of pharmacological treatments.
- Graphical analysis: Mean BMI trajectories were plotted for each major pharmacological subgroup over the ten-week period.
- Generalized linear model (GLM):
  ∘ A generalized linear model (Gaussian family) was applied, using ΔBMI and AMI as dependent variables in separate models.
  ∘ Independent variables: binary indicators for each specific drug and pharmacological class.
  ∘ Model outputs included regression coefficients with standard errors, z-values, 95% confidence intervals, and p-values.

All statistical analyses were performed using R version 4.4.0 (20) and the tidyverse, ggplot2, and lme4 packages (20).

### Model interpretation

Two GLMs were produced:

- The primary model tested the association between pharmacological treatments and BMI change (ΔBMI).
- A secondary model explored the effect of the same treatments on changes in the AMI as supportive information.

Regression coefficients were interpreted as follows:

- Positive coefficient: associated with a greater BMI increase or higher AMI.
- Negative coefficient: associated with a smaller BMI increase or a reduction in morbidity.

The GLM coefficients reflect adjusted associations, controlling for potential confounders such as age and gender, whereas the mean ΔBMI and AMI values reported in the summary table represent unadjusted raw means for each pharmacological subgroup. Analyses were conducted at two complementary levels: (i) pharmacological class, representing the primary inferential framework of the study, and (ii) individual compounds, reported descriptively and exploratorily to reflect real-world prescribing practices and to generate hypotheses for future studies. Results related to individual medications prescribed to small subsamples were not intended to support definitive conclusions. Accordingly, **Table 1** is structured to distinguish class-level summaries (Section A) from compound-level descriptive findings (Section B).

**Table 1:** Changes in body mass index (ΔBMI) and psychopathological symptom burden (AMI) according to psychopharmacological treatment, by pharmacological class (Section A) and individual compounds (Section B).

Section A. Pharmacological classes				
Pharmacological class	N patients (%)		Mean ΔBMI	Mena AMI
Antidepressants	113 (80.9%)		≈ 2.36	≈ 2.17
Mood Stabilizers	45 (35.4%)		≈ 1.86	≈ 1.93
Antypshicotics	95 (74.8%)		≈ 2.42	≈ 2.15
Benzodiazepines	60 (47.2%)		≈ 2.18	≈ 1.98

Section B. Individual compounds				
Class	Drug	N (%)	Mean ΔBMI	Mean AMI
Antidepressant	Duloxetine	2 (1.6%)	0.65	2.50
Antidepressant	Sertraline	62 (48.8%)	2.72	2.20
Antidepressant	Paroxetine	4 (3.1%)	2.05	2.05
Antidepressant	Escitalopram	7 (5.5%)	3.10	2.01
Antidepressant	Vortioxetine	7 (5.5%)	1.97	2.10
Antidepressant	Mianserine	1 (0.8%)	1.50	3.00
Antidepressant	Fluoxetine	6 (4.7%)	1.60	1.95
Antidepressant	Fluvoxamine	17 (13.4%)	2.35	2.31
Antidepressant	Clomipramine	2 (1.6%)	-0.75	1.50
Antidepressant	Venlafaxine	5 (3.9%)	1.24	1.94
Mood stabilizer	Carbamazepine	28 (22.0%)	1.73	1.93
Mood stabilizer	Valproic acid	2 (1.6%)	2.65	1.85
Mood stabilizer	Lithium Carbonate	6 (4.7%)	3.47	2.47
Mood stabilizer	Lithium Sulfate	5 (3.9%)	2.10	1.70
Mood stabilizer	Oxcarbazepine	1 (0.8%)	-0.30	1.30
Mood stabilizer	Lamotrigine	2 (1.6%)	1.15	1.70
Mood stabilizer	Gabapentin	1 (0.8%)	1.30	2.00
Antipsychotic	Haloperidol	5 (3.9%)	2.48	2.10
Antipsychotic	Promazine	2 (1.6%)	3.70	2.90
Antipsychotic	Olanzapine	36 (28.3%)	2.13	2.19
Antipsychotic	Aripiprazole	39 (30.7%)	2.61	2.12
Antipsychotic	Quetiapine	2 (1.6%)	0.70	1.50
Antipsychotic	Lurasidone	8 (6.3%)	2.14	2.04
Antipsychotic	Cariprazine	3 (2.4%)	2.87	2.33
Antipsychotic	Risperidone	1 (0.8%)	3.00	1.80
Antipsychotic	Levosulpiride	1 (0.8%)	-0.30	1.20
Benzodiazepines	Delorazepam	46 (36.2%)	2.27	2.15
Benzodiazepines	Alprazolam	5 (3.9%)	1.76	1.72
Benzodiazepines	Diazepam	4 (3.1%)	2.23	1.50
Benzodiazepines	Clonazepam	2 (1.6%)	0.85	1.00
Benzodiazepines	Lorazepam	3 (2.4%)	1.77	2.10

Reports results by pharmacological class and represents the primary analytical framework of the study. Due to polypharmacy, patients may be represented in more than one pharmacological class. Accordingly, class-level values represent unadjusted, descriptive summaries and do not reflect mutually exclusive treatment groups.

BMI, Body Mass Index; ΔBMI, Change in Body Mass Index; AMI, Average Morbidity Index; N = number of patients exposed at least once to the drug class during hospitalization.

Reports results for individual medications for descriptive and exploratory purposes only, reflecting real-world prescribing patterns. Findings related to compounds prescribed to very small subsamples (N < 5) should be interpreted with particular caution and are intended to be hypothesis-generating.

## Results

**Figure 1:**
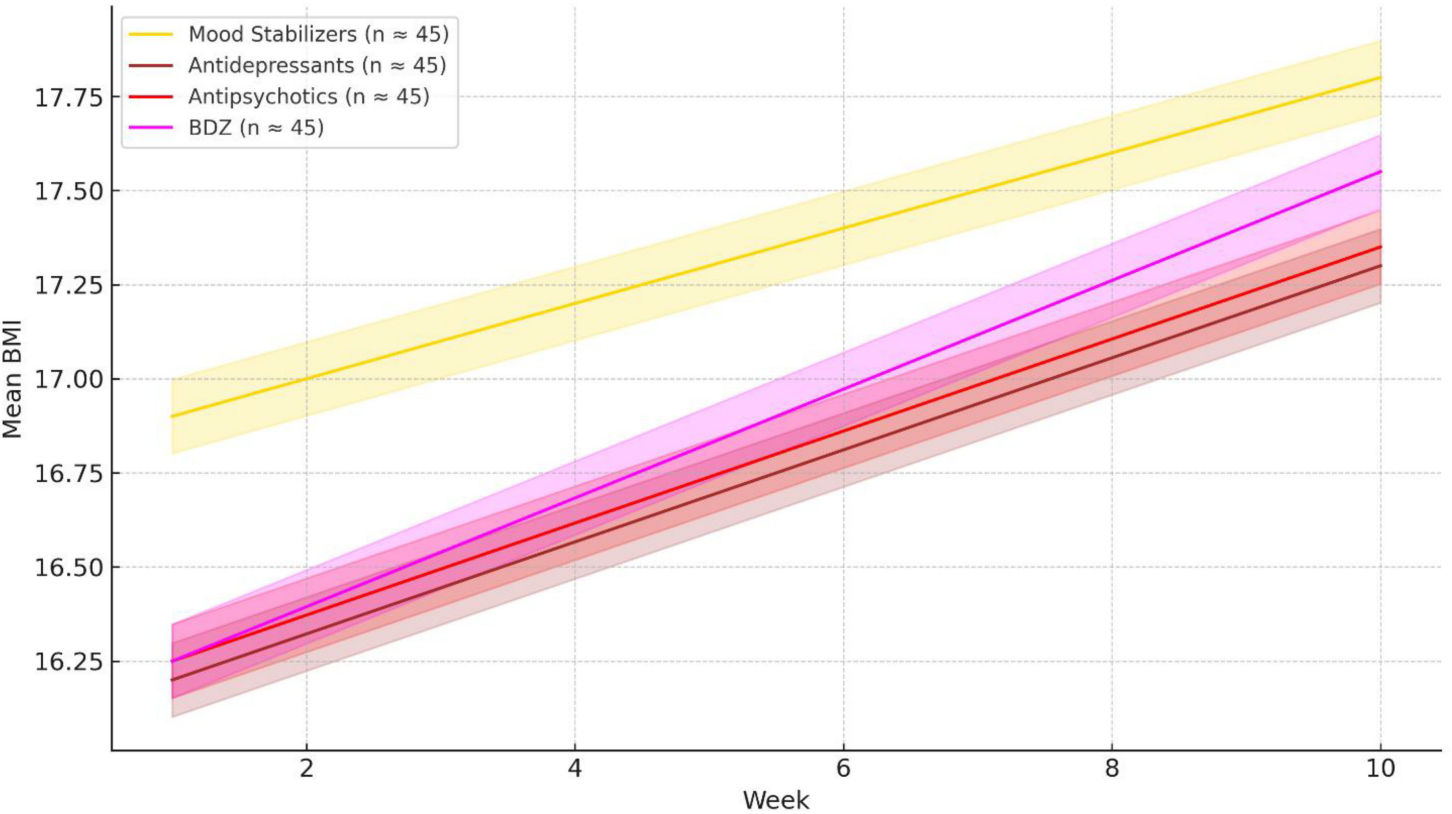
Mean body mass index (BMI) trajectories over the 10-week inpatient treatment period, stratified by psychopharmacological class. Solid lines represent weekly mean BMI for patients treated with mood stabilizers (gold), antidepressants (brown), antipsychotics (red), or benzodiazepines (magenta). Shaded areas indicate the 95% confidence intervals for each group. Patients receiving mood stabilizers showed a more moderate BMI increase compared to other drug classes. Approximate sample sizes: Mood Stabilizers (n = 45), Antidepressants (n = 62), Antipsychotics (n = 91), Benzodiazepines (n = 57).

**Figure 2:**
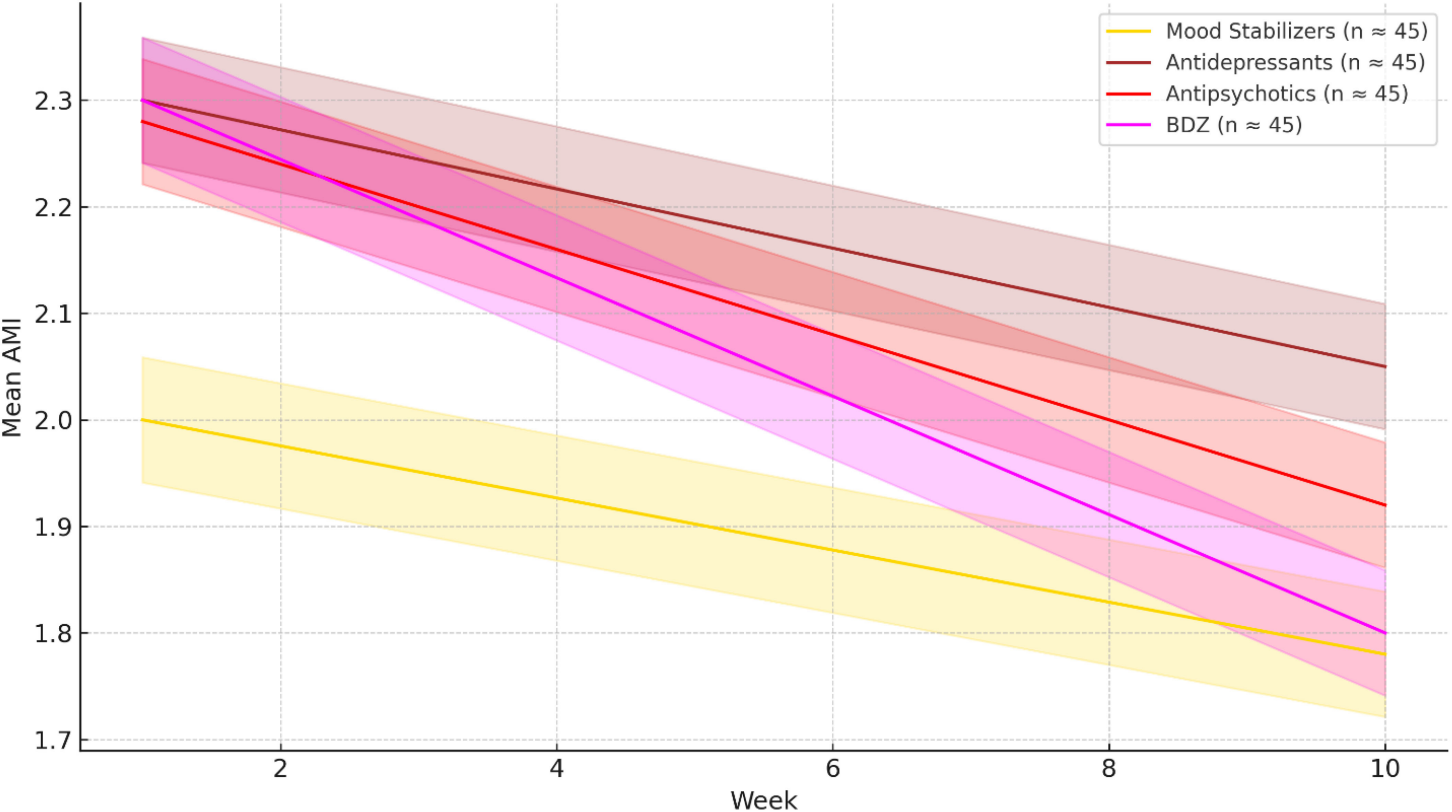
Mean Average Morbidity Index (AMI) trajectories by psychopharmacological class over the same 10-week period. Solid lines show weekly mean AMI values; shaded bands represent 95% confidence intervals. Lower AMI scores indicate greater improvement in psychopathological symptom burden. Compared to other drug classes, mood stabilizers and antipsychotics were associated with more pronounced reductions in AMI over time. Approximate sample sizes: Mood Stabilizers (n = 45), Antidepressants (n = 62), Antipsychotics (n = 91), Benzodiazepines (n = 57).

A total of 127 patients with a mean age of 18.7 years (SD ± 5.5) were included in the final analysis. Most of the sample was diagnosed with restrictive anorexia nervosa (AN-r, 73.2%), followed by binge-purge subtype anorexia nervosa (AN-bp, 15.7%) and bulimia nervosa (BN, 10.2%). At admission, the mean BMI was 16.3 (SD ± 2.9) and increased significantly to 18.6 (SD ± 2.2) at discharge, resulting in an average ΔBMI of +2.3 (SD ± 1.7). The mean AMI score showed a progressive reduction during the ten-week period, indicating an overall symptomatic improvement (mean reduction of about 0.4 points) (**Table 2**). Primary results are presented at the level of pharmacological classes. Findings related to individual compounds are reported to illustrate within-class variability and should be interpreted as exploratory. **Table 1** summarizes psychopharmacological treatments and associated changes in BMI (ΔBMI) and psychopathological symptom burden (AMI). Results are presented by pharmacological class (Section A), which represents the primary analytical framework of the study, and by individual compounds (Section B), which are reported for descriptive and exploratory purposes. Generalized Linear Models (GLM) were used to investigate the association between pharmacological treatments and changes in BMI and AMI scores, controlling for age and gender. For BMI trajectories, the GLM showed that Carbamazepine (Coef. = -0.96, p = 0.0055) and Lithium Sulfate (Coef. = -1.70, p = 0.0231) were significantly associated with a smaller increase in BMI over time, suggesting a weight-stabilizing effect in patients treated with mood stabilizers. In contrast, the use of Diazepam (Coef. = +2.06, p = 0.0208) was significantly associated with greater weight gain. Duloxetine (Coef. = -2.95, p = 0.0440) and Paroxetine (Coef. = -2.31, p = 0.0534) also showed a trend towards mitigating BMI increase among antidepressants (**Table 3**). Regarding the AMI, the model indicated that several antidepressants Sertraline (Coef. = +0.41, p = 0.0096), Escitalopram (Coef. = +0.37, p = 0.0423), Vortioxetine (Coef. = +0.42, p = 0.0088), Fluvoxamine (Coef. = +0.50, p = 0.0032), and Venlafaxine (Coef. = +0.44, p = 0.0358) were significantly associated with higher AMI scores, indicating less pronounced symptomatic improvement compared to other groups (**Table 1**). In contrast, several atypical antipsychotics Olanzapine, Aripiprazole, Quetiapine, Lurasidone, Cariprazine, Levosulpiride showed negative coefficients with significant p-values (all p< 0.05), suggesting a stronger contribution to psychopatological symptom reduction (**Table 4**). The graphical analysis further supports these findings. The BMI trend plot shows that patients receiving mood stabilizers exhibited the slowest increase in BMI, while those treated with benzodiazepines showed a steeper rise (**Figure 1**). The AMI trend plot illustrates that the overall psychopatological symptom severity decreased more markedly in patients treated with antipsychotics and mood stabilizers compared to those on antidepressants or benzodiazepines alone (**Figure 2**). Age showed a small but significant positive effect on AMI scores (Coef. = +0.013, p = 0.0144), while gender did not significantly affect BMI or AMI outcomes in this sample.

**Table 2:** Baseline characteristics of the total sample and distribution of psychopharmacological treatments during the inpatient program.

Total sample 127	
Variable	Mean ± SD / N (%)
Age (years)	18.7 ± 5.5
BMI at admission	16.3 ± 2.9
BMI at discharge	18.6 ± 2.2
ΔBMI (discharge - admission)	2.3 ± 1.7
AN-r	93 (73.2%)
AN-bp	20 (15.7%)
BN	13 (10.2%)
AN.r	1 (0.8%)
Duloxetine	2 (1.6%)
Sertraline	62 (48.8%)
Paroxetine	4 (3.1%)
Escitalopram	7 (5.5%)
Vortioxetine	7 (5.5%)
Mianserine	1 (0.8%)
Fluoxetine	6 (4.7%)
Fluvoxamine	17 (13.4%)
Clomipramine	2 (1.6%)
Venlafaxine	5 (3.9%)
Carbamazepine	45 (35.4%)
Valproic acid	28 (22.0%)
Lithium Carbonate	2 (1.6%)
Lithium Sulfate	6 (4.7%)
Oxcarbazepine	5 (3.9%)
Lamotrigine	1 (0.8%)
Gabapentin	2 (1.6%)
Haloperidol	1 (0.8%)
Promazine	6 (4.7%)
Olanzapine	5 (3.9%)
Aripiprazole	2 (1.6%)
Quetiapine	91 (71.7%)
Lurasidone	36 (28.3%)
Cariprazine	39 (30.7%)
Risperidone	2 (1.6%)
Levosulpiride	8 (6.3%)
Delorazepam	3 (2.4%)
Alprazolam	1 (0.8%)
Diazepam	1 (0.8%)
Clonazepam	57 (44.9%)
Lorazepam	46 (36.2%)
Duloxetine	5 (3.9%)
Sertraline	4 (3.1%)
Paroxetine	2 (1.6%)
Escitalopram	3 (2.4%)

BMI, Body Mass Index; AN-r, Anorexia Nervosa, Restricting type; AN-bp Anorexia Nervosa, Binge-Eating/Purging Type; BN, Bulimia Nervosa; ΔBMI, Change in Body Mass Index.

**Table 3:** Generalized Linear Model (GLM) for ΔBMI.

Variable	Coef.	Std.Err.	z	P>|z|	95% CI
Antidepressant	2.0512	1.0017	2.0478	0.0406	[0.088, 4.0144]
Duloxetine	-2.9513	1.4655	-2.0139	0.044	[-5.8237, -0.079]
Paroxetine	-2.3107	1.1961	-1.9318	0.0534	[-4.6551, 0.0337]
Carbamazepine	-0.9613	0.3459	-2.779	0.0055	[-1.6393, -0.2833]
Lithium Sulfate	-1.7018	0.749	-2.272	0.0231	[-3.1698, -0.2337]
Delorazepam	0.5477	0.3027	1.8092	0.0704	[-0.0456, 1.1411]
Diazepam	2.0557	0.8896	2.3109	0.0208	[0.3122, 3.7993]

ΔBMI, change in Body Mass Index ; Coef., coefficients; Std.Err., standard errors; P>|z|, z-values; p-values; 95% CI, 95% confidence intervals.

**Table 4:** Generalized mixed model AMI.

Variable	Coef.	Std.Err.	z	P>|z|	[0.025 - 0.975]
Intercept	0.249	0.339	0.734	0.4630	[-0.415, 0.913]
Antidepressant	-0.255	0.159	-1.607	0.1080	[-0.566, 0.056]
Duloxetine	0.544	0.241	2.261	0.0238	[0.072, 1.015]
Sertraline	0.408	0.158	2.589	0.0096	[0.099, 0.717]
Paroxetine	0.352	0.191	1.840	0.0658	[-0.023, 0.727]
Escitalopram	0.373	0.184	2.031	0.0423	[0.013, 0.734]
Vortioxetine	0.418	0.160	2.618	0.0088	[0.105, 0.730]
Mianserine	0.620	0.259	2.392	0.0168	[0.112, 1.128]
Fluoxetine	0.327	0.181	1.812	0.0700	[-0.027, 0.682]
Fluvoxamine	0.500	0.170	2.944	0.0032	[0.167, 0.833]
Clomipramine	-0.060	0.237	-0.255	0.7987	[-0.524, 0.403]
Venlafaxine	0.437	0.208	2.100	0.0358	[0.029, 0.845]
Mood stabilizers	-0.212	0.140	-1.507	0.1318	[-0.487, 0.064]
Carbamazepine	0.074	0.143	0.513	0.6079	[-0.207, 0.354]
Valproic acid	0.175	0.234	0.746	0.4558	[-0.284, 0.634]
Lithium Carbonate	0.109	0.178	0.614	0.5390	[-0.240, 0.458]
Lithium Sulfate	-0.146	0.184	-0.794	0.4275	[-0.506, 0.214]
Oxcarbazepine	0.042	0.287	0.147	0.8829	[-0.521, 0.606]
Lamotrigine	0.242	0.253	0.959	0.3374	[-0.253, 0.738]
Gabapentin	-0.018	0.312	-0.059	0.9532	[-0.629, 0.593]
Antipsichotics	-0.122	0.258	-0.473	0.6359	[-0.629, 0.384]
Haloperidol	0.051	0.229	0.224	0.8229	[-0.397, 0.500]
Promazine	0.460	0.312	1.472	0.1409	[-0.152, 1.071]
Antipsychotics II	0.581	0.261	2.226	0.0260	[0.069, 1.093]
Olanzapine	-0.576	0.268	-2.153	0.0313	[-1.101, -0.052]
Aripiprazole	-0.575	0.261	-2.201	0.0278	[-1.087, -0.063]
Quetiapine	-0.661	0.308	-2.146	0.0319	[-1.265, -0.057]
Lurasidone	-0.706	0.276	-2.559	0.0105	[-1.246, -0.165]
Cariprazine	-0.743	0.320	-2.320	0.0203	[-1.370, -0.115]
Risperidone	-0.623	0.348	-1.791	0.0732	[-1.305, 0.059]
Levosulpiride	-0.900	0.360	-2.501	0.0124	[-1.605, -0.195]
BDZ	0.125	0.230	0.546	0.5851	[-0.325, 0.576]
Delorazepam	-0.150	0.228	-0.660	0.5091	[-0.597, 0.296]
Alprazolam	-0.195	0.271	-0.717	0.4733	[-0.727, 0.337]
Diazepam	-0.333	0.266	-1.251	0.2110	[-0.855, 0.189]
Clonazepam	-0.252	0.208	-1.211	0.2261	[-0.660, 0.156]
Lorazepam	-0.036	0.303	-0.118	0.9064	[-0.630, 0.558]
Gender	0.076	0.163	0.467	0.6408	[-0.243, 0.395]
Age	0.013	0.005	2.446	0.0144	[0.003, 0.023]

## Discussion

The present prospective observational study suggests that different psychopharmacological agents may exert distinct influences on both nutritional recovery and short-term psychopatological symptom trajectories in patients with eating disorders. By measuring BMI and morbidity indices weekly and in real time, this work provides a dynamic perspective on how medication regimens interact with clinical evolution during intensive inpatient treatment. Mood stabilizers, antidepressants, antipsychotics, and benzodiazepines demonstrated divergent prospective associations with both BMI and AMI patterns, offering clinically relevant signals that could support more personalized treatment strategies.

A core innovative aspect of the study is the adaptation of a life-chart framework, operationalized here through the AMI, to monitor weekly fluctuations in psychopathology alongside nutritional progress. This approach extends the well-established Life Chart Methodology used in mood disorders to a population in which psychopatological symptom variability is rarely captured with comparable temporal resolution. This aligns with recent advances in digital phenotyping and intensive longitudinal assessment, which emphasize that short-interval monitoring captures psychopatological symptom fluctuations that are often missed by traditional point-based evaluations (21). The AMI offers a clinically feasible analogue to these digital approaches in settings where continuous data capture is not available. By integrating structured weekly psychopathology ratings with BMI trajectories, the AMI provides a nuanced representation of how symptom burden and weight restoration co-evolve during treatment. Importantly, although inpatient treatment settings effectively limit overt eating disorder behaviors, internal psychopathological symptoms often persist and fluctuate during hospitalization. The AMI was therefore intended to capture this internal symptom burden, which remains clinically relevant despite behavioral containment and may influence treatment response and relapse vulnerability. To our knowledge, this is among the first applications of a structured morbidity index to EDs, highlighting the value of chart-derived, time-series data for informing individualized adjustments in real-world clinical settings. While life-chart methodologies are widely used in bipolar disorder and related conditions (22), their systematic application to EDs remains limited (23). The present findings illustrate how combining longitudinal morbidity indices with weekly BMI monitoring can illuminate potential medication-specific influences on both symptomatic and nutritional outcomes. Pharmacological treatment of EDs remains largely off-label, as no medications are currently approved for core AN symptoms (13). SSRIs, particularly fluoxetine, have demonstrated benefits in BN and BED (24), but evidence for weight restoration or relapse prevention in AN remains weak (4, 12, 25–29). Atypical antipsychotics, especially olanzapine, have been explored as adjunctive treatments for weight gain in AN, with highly variable and often modest results (26–28). Our findings are broadly consistent with the results of the largest randomized controlled trial of olanzapine in AN (9), which reported significantly greater weight gain in patients receiving olanzapine compared with placebo, with limited effects on core eating disorder psychopathology. Extending these findings to a real-world inpatient context, our data suggest that olanzapine may contribute not only to weight restoration but also to reductions in overall psychopathological burden, as captured by the AMI, during intensive treatment.

Mood stabilizers have received even less attention, typically prescribed in the presence of comorbid affective instability or impulse-control difficulties (30). Benzodiazepines are often used transiently to manage acute anxiety or sleep disturbance, yet their potential influence on BMI trajectories, particularly in younger patients, is poorly understood. These findings can be further contextualized in light of current international treatment recommendations. The 2023 update of the World Federation of Societies of Biological Psychiatry (WFSBP) guidelines emphasize that no pharmacological agents are approved for the core treatment of anorexia nervosa, and that medications should be considered only as adjuncts to nutritional rehabilitation and psychotherapy, primarily to address comorbid symptoms or specific clinical targets (6). In particular, the guidelines identify atypical antipsychotics, especially olanzapine, the agents with the most consistent, albeit modest, evidence for supporting weight gain, while highlighting limited and inconsistent evidence for antidepressants and the largely exploratory role of mood stabilizers. The patterns observed in our real-world inpatient sample broadly align with these recommendations, supporting cautious, individualized use of psychotropic medications rather than class-wide assumptions of efficacy.

Against this background, our study provides real-world insights into how commonly used psychotropic agents may shape both nutritional and symptomatic progression in individuals with severe EDs, predominantly AN. The signal suggesting that mood stabilizers may support a balance between weight gain and containment of psychopatological symptom fluctuation could inform future trials aimed at optimizing multidrug strategies for treatment-resistant AN, a group with limited evidence-based options once first-line interventions fail. As shown in **Table 1A**, class-level patterns were consistent with the adjusted associations observed in the regression models, whereas compound-level findings reported in **Table 1B** should be interpreted as exploratory. The observation that diazepam was associated with greater BMI gain but did not confer parallel symptomatic benefit underscores the importance of careful benefit-risk assessment when prescribing sedatives in this vulnerable population. Our sample, predominantly young women with AN-r, mirrors the demographic profile typically observed in specialized inpatient settings (3), enhancing ecological validity despite limiting generalizability to broader ED populations. This methodological framework also offers a replicable strategy for capturing dynamic changes in psychopatological symptom severity, a dimension often missed by categorical or point-in-time assessments. Integrating AMI-based monitoring with nutritional indicators may help elucidate how specific pharmacological combinations influence core psychopathology over time, informing more nuanced and individualized treatment approaches. The magnitude of BMI increase observed in this study should be interpreted in the context of medically supervised inpatient care, where weight restoration is intentionally gradual, particularly in patients with severe malnutrition. Early phases of hospitalization often prioritize medical stabilization and psychological containment over rapid weight gain, and substantial inter-individual variability further attenuates mean short-term BMI changes in naturalistic samples.

## Limitations

Several limitations should be acknowledged. The sample consisted predominantly of young females with AN-r reflecting the demographic profile typically treated in specialized inpatient programs. While this enhances ecological validity, it limits generalizability to broader ED populations, including older individuals, males, and patients with binge–purge presentations. Although a small proportion of patients with bulimia nervosa was included, the cohort was predominantly composed of individuals with anorexia nervosa. Given that weight restoration is not a primary treatment goal in BN, BMI-based findings should be interpreted mainly in relation to the AN inpatient population. The naturalistic and non-randomized design does not permit causal inference, and treatment allocation is likely to have reflected clinician judgment and baseline illness severity. Similarly, the single-center setting ensures consistency in assessment procedures and treatment protocols but may reduce generalizability to services with different clinical practices. Although the AMI provides a structured measure of weekly psychopatological symptom burden, its use in eating disorders requires further validation and standardization to facilitate comparisons across studies and to support potential meta-analytic integration (27). Future research should examine its correspondence with established psychopathology scales and assess inter-rater reliability to strengthen its psychometric foundations. Despite prospective data collection using a predefined protocol, naturalistic settings remain susceptible to variability in documentation and occasional missing data. No *a priori* power calculation was feasible due to the exploratory nature of the study; accordingly, subgroup analyses, particularly for less frequently prescribed medications, should be interpreted as hypothesis-generating rather than definitive. Findings related to compounds prescribed in very small numbers should be interpreted with particular caution. Accordingly, compound-level findings are intended to be hypothesis-generating and should not be interpreted independently of their pharmacological class. Finally, the ten-week follow-up captures only short-term clinical dynamics, and longer prospective studies are needed to evaluate the durability of treatment effects and relapse risk.

## Conclusion

In summary, this prospective real-world study highlights the value of integrating life-chart approaches, such as the AMI, with routine nutritional monitoring to elucidate the complex interplay between pharmacotherapy, psychopatological symptom evolution, and weight restoration in eating disorders. By combining weekly morbidity ratings with systematic BMI assessment, we provide a dynamic characterization of early treatment trajectories and identify medication-specific patterns that may support more individualized clinical decision-making. Our findings indicate that certain mood stabilizers may help modulate psychopatological symptom fluctuations while facilitating nutritional rehabilitation, whereas some sedative agents appear to influence weight gain without conferring parallel improvements in psychopathology. Antidepressants and atypical antipsychotics also showed differentiated effects, underscoring the need for tailored pharmacological strategies rather than broad class-level assumptions. These preliminary signals warrant confirmation in larger, controlled prospective studies aimed at clarifying medication effects and informing evidence-based pharmacotherapeutic pathways for treatment-resistant eating disorders. Given the continued absence of formally approved pharmacological treatments for AN, innovative real-world approaches such as the one adopted in this study remain essential for advancing more precise, mechanism-informed interventions for this highly vulnerable population.

## Data Availability

The raw data supporting the conclusions of this article will be made available by the authors, without undue reservation.

## References

[1] Hay P . Current approach to eating disorders: A clinical update. Intern Med J. (2020) 50:24–9. doi: 10.1111/imj.14691, PMID: 31943622 PMC7003934

[2] National Institute for Health and Care Excellence . Eating disorders (Quality standard QS175) (2018). Available online at: https://www.nice.org.uk/guidance/qs175 (Accessed November 12, 2025).

[3] Merikangas KR He JP Burstein M Swanson SA Avenevoli S Cui L . Lifetime prevalence of mental disorders in U.S. adolescents. J Am Acad Child Adolesc Psychiatry. (2010) 49:980–9. doi: 10.1016/j.jaac.2010.05.017, PMID: 20855043 PMC2946114

[4] Miskovic-Wheatley J Bryant E Ong SH Vatter S Le A Touyz S . Eating disorder outcomes: Findings from a rapid review of over a decade of research. J Eat Disord. (2023) 11:85. doi: 10.1186/s40337-023-00801-3, PMID: 37254202 PMC10228434

[5] Krug I Dang AB Lu E Ooi WL Portingale J Miles S . A narrative review on the neurocognitive profiles in eating disorders and higher weight individuals: Insights for targeted interventions. Nutrients. (2024) 16:4418. doi: 10.3390/nu16244418, PMID: 39771039 PMC11677587

[6] Himmerich H Lewis YD Conti C Mutwalli H Karwautz A Sjögren JM . World Federation of Societies of Biological Psychiatry (WFSBP) guidelines update 2023 on the pharmacological treatment of eating disorders. World J Biol Psychiatry. (2023) 24:643–706. doi: 10.1080/15622975.2023.2179663, PMID: 37350265

[7] Pruccoli J Bergonzini L La Tempa A Parmeggiani A . Antipsychotics in the treatment of children and adolescents with anorexia nervosa: A systematic review. Biomedicines. (2022) 10:3167. doi: 10.3390/biomedicines10123167, PMID: 36551922 PMC9775317

[8] Said O Stringer D Sengun Filiz E Mutwalli H Bektas S Akkese MN . Olanzapine for young people with anorexia nervosa (OPEN): Results of a feasibility study. Eur Eat Disord Rev. (2024) 32:532–46. doi: 10.1002/erv.3060, PMID: 38299859

[9] Attia E Steinglass JE Walsh BT Wang Y Wu P Schreyer C . Olanzapine versus placebo in adult outpatients with anorexia nervosa: A randomized clinical trial. Am J Psychiatry. (2019) 176:449–56. doi: 10.1176/appi.ajp.2018.18101125, PMID: 30654643 PMC7015155

[10] Bindseil I Stutzman DL Schiel MA Sheffield K Hagman J . Utility of aripiprazole in the treatment of anorexia nervosa in children and adolescents: A retrospective matched cohort study. J Child Adolesc Psychopharmacol. (2025) 35:471–8. doi: 10.1089/cap.2025.0013, PMID: 40537086

[11] Andries A Frystyk J Flyvbjerg A Støving RK . Dronabinol in severe, enduring anorexia nervosa: A randomized controlled trial. Int J Eat Disord. (2014) 47:18–23. doi: 10.1002/eat.22173, PMID: 24105610

[12] Walsh BT Kaplan AS Attia E Olmsted M Parides M Carter JC . Fluoxetine after weight restoration in anorexia nervosa: A randomized controlled trial. JAMA. (2006) 295:2605–12. doi: 10.1001/jama.295.22.2605, PMID: 16772623

[13] Rodan SC Bryant E Le A Maloney D Touyz S McGregor IS . Pharmacotherapy, alternative and adjunctive therapies for eating disorders: Findings from a rapid review. J Eat Disord. (2023) 11:112. doi: 10.1186/s40337-023-00833-9, PMID: 37415200 PMC10327007

[14] NICE/APA . Guideline on psychopharmacology for anorexia nervosa. In: Annotated clinical guidelines for eating disorders. National Institute for Health and Care Excellence; American Psychiatric Association, London (2024). p. 45–67.

[15] Besjes MJ Mares SHW van Elburg AA Spijker J . The efficacy of pharmacological treatment of depression in anorexia nervosa and underweight patients: A systematic review. Eur Eat Disord Rev. (2025) 34:55–70. doi: 10.1002/erv.70008, PMID: 40643320 PMC12694688

[16] Márquez MC Sánchez JM Salazar AM Martínez CV Valderrama F Rojas-Gualdrón DF . Efficacy and safety of antipsychotics and antidepressants in the treatment of anorexia nervosa: A systematic review. Rev Colomb Psiquiatr (Engl Ed). (2021) 51:227–35. doi: 10.1016/j.rcpeng.2022.08.007, PMID: 36085125

[17] Bauschka M Watters A Blalock D Farooq A Mehler P Gibson D . Atypical antipsychotic use does not impact weight gain for individuals with extreme anorexia nervosa: A retrospective case-control study. J Eat Disord. (2023) 11:215. doi: 10.1186/s40337-023-00941-6, PMID: 38057934 PMC10699020

[18] Toppino F Martini M Longo P Caldas I Delsedime N Lavalle R . Inpatient treatments for adults with anorexia nervosa: A systematic review of the literature. Eat Weight Disord. (2024) 29:38. doi: 10.1007/s40519-024-01665-5, PMID: 38767754 PMC11106202

[19] Suppes T Leverich GS Keck PE Nolen WA Denicoff KD Altshuler LL . The Stanley Foundation bipolar treatment outcome network. II. Demographics and illness characteristics of the first 261 patients. J Affect Disord. (2001) 67:45–59. doi: 10.1016/S0165-0327(01)00432-3, PMID: 11869752

[20] R Core Team . R: A language and environment for statistical computing. Vienna: R Foundation for Statistical Computing (2025). Available online at: https://www.R-project.org/ (Accessed November 13, 2025).

[21] Linardon J Torous J . Integrating artificial intelligence and smartphone technology to enhance personalized assessment and treatment for eating disorders. Int J Eat Disord. (2025) 58:1415–24. doi: 10.1002/eat.24468, PMID: 40396625 PMC12336790

[22] Bauer M Glenn T Alda M Grof P Bauer R Ebner-Priemer UW . Longitudinal digital mood charting in bipolar disorder: Experiences with ChronoRecord over 20 years. Pharmacopsychiatry. (2023) 56:182–7. doi: 10.1055/a-2156-5667, PMID: 37678394 PMC10484643

[23] Post RM . The new news about lithium: An underutilized treatment in the United States. Neuropsychopharmacology. (2018) 43:1174–9. doi: 10.1038/npp.2017.238, PMID: 28976944 PMC5854802

[24] Norris ML Spettigue W Buchholz A Henderson KA Gomez R Maras D . Olanzapine use for the adjunctive treatment of adolescents with anorexia nervosa. J Child Adolesc Psychopharmacol. (2011) 21:213–20. doi: 10.1089/cap.2010.0131, PMID: 21510781 PMC3111870

[25] Attia E Steinglass JE . Pharmacologic treatment of anorexia nervosa: Where do we go from here? Int J Eat Disord. (2017) 50:329–32. doi: 10.1002/eat.20133, PMID: 15852322

[26] Bacaltchuk J Hay P . Antidepressants versus placebo for people with bulimia nervosa. Cochrane Database Syst Rev. (2003) 2003:CD003391. doi: 10.1002/14651858.CD003391, PMID: 14583971

[27] McDonald CE Rossell SL Phillipou A . The comorbidity of eating disorders in bipolar disorder and associated clinical correlates characterized by emotion dysregulation and impulsivity: A systematic review. J Affect Disord. (2019) 259:228–43. doi: 10.1016/j.jad.2019.08.070, PMID: 31446385

[28] Mitchell JE Agras S Wonderlich S . Treatment of bulimia nervosa: Where are we and where are we going? Int J Eat Disord. (2013) 46:478–85. doi: 10.1002/eat.20343, PMID: 17080448

[29] Fluoxetine Bulimia Nervosa Collaborative Study Group . Fluoxetine in the treatment of bulimia nervosa: A multicenter, placebo-controlled, double-blind trial. Arch Gen Psychiatry. (1992) 49:139–47. doi: 10.1001/archpsyc.1992.01820020059008 1550466

[30] Guarda AS Schreyer CC Boersma GJ Tamashiro KL Moran TH . Anorexia nervosa as a motivated behavior: Relevance of anxiety, stress, fear, and learning. Physiol Behav. (2015) 152:466–72. doi: 10.1016/j.physbeh.2015.04.007, PMID: 25846837

